# Bayesian adaptive sampling: A smart approach for affordable germination phenotyping

**DOI:** 10.1016/j.plaphe.2025.100067

**Published:** 2025-06-21

**Authors:** Félix Mercier, Nizar Bouhlel, Angelina El Ghaziri, Joseph Ly Vu, Julia Buitink, David Rousseau

**Affiliations:** a LARIS, Université d’Angers, Angers, France; b IRHS, UMR1345, INRAE, Université d’Angers, Institut Agros, Beaucouzé, France

**Keywords:** Low Cost phenotyping, Seed Germination, Adaptive Sampling, Bayesian methods

## Abstract

Digital phenotyping is rapidly advancing, generating increasing amounts of data, particularly in the case of temporal monitoring. We propose an adaptive sampling method that optimizes sampling, thereby reducing costs associated with data production, processing, and storage. The proposed method is based on Bayesian inference, which utilizes previous measurements, historical data, and an expected model. Five Bayesian methods are assessed in this study: Important sampling (IS), Markov chain Monte-Carlo (MCMC), Gaussian process (GP), Extended Kalman filtering (EKF) and Sampling Importance Resampling particle filtering (SIR-PF). We test these five Bayesian sampling methods for the monitoring of germination rate in terms of compression, distortion and computation cost. The best trade-off is found by the MCMC method, which offers a compression rate of 0.2 with very little distortion. GP offers the most unbiased parameter estimation and the capability to adapt to various germination speeds. It also has reasonable computational times.

## 1 Introduction

Over the past twenty years, plant imaging combined with computer vision has gained significant attention [1,2]. This field plays a crucial role in biology and agriculture by enabling objective quantification at observational scales beyond human perception, whether due to spectral, spatial, or temporal limitations. One of the main challenges in plant imaging is the continuous growth of plants and the need to analyze large populations for statistically meaningful results. Monitoring plant development over long periods generates vast amounts of data, which requires extensive processing. A common approach is to use a fixed sampling rate for data collection. However, plant growth varies according to species, genotype, and environmental conditions. Additionally, plant development does not follow a simple linear timescale, making it difficult to determine an optimal fixed sampling rate. To address this, adaptive sampling methods provide a more efficient alternative by dynamically adjusting data collection and preventing unnecessary data overload.

In this study, we focus on a practical application of plant imaging: monitoring seed germination kinetics through time-lapse RGB images using adaptive sampling. The field of adaptive temporal sampling includes various approaches [3], such as Bayesian methods and Gaussian Processes, which allow observations to be selected based on prior measurements. Since these observations are modeled as random variables, adapting the sampling strategy is particularly important when dealing with non-linear processes. This need has been recognized in several domains, including mobile sensor networks [4], robotics [5], the Internet of Things [6], and plant phenotyping [7], where Gaussian Processes have been previously applied. In this work, we present a Bayesian approach based on online uncertainty estimation as recently introduced by our group in [8]. To further extend and evaluate the effectiveness of this approach, we compare five Bayesian estimation techniques for sampling non-linear germination model in adaptive manner. In addition to Importance Sampling (IS) and Markov Chain Monte Carlo (MCMC), performed in [8], the Gaussian Process (GP) method is improved and enhanced in this study. Moreover, this study also introduces two sequential estimation methods (not included in [8]): the Extended Kalman Filter (EKF) and the Sequential Importance Resampling (SIR), expanding the range of tools available for adaptive sampling.

As additional elements, not included in [8], we provide the codes and data to perform the computation and discuss the convergence of the process: The code is available at the following address: https://github.com/Fatryuk/BayesianAdaptivSampling.git. To address the issue of adaptive sampling, we apply these methods to seed germination across different genotypes, and we examine key aspects, such as the compression-distortion trade-off, computational efficiency, and potential bias. The paper is organized as follows. In Section II, we introduce the materials and methods. Five Bayesian methods are briefly presented, with a detailed mathematical and algorithmic description provided separately in the supplementary material. The simulated and real data used for the evaluation, along with the experimental settings, are also described. Section III details the performance analysis of the proposed methods in terms of compression-distortion rate, computational efficiency, and potential bias. Finally, the discussion and some concluding remarks close up this paper.

## 2 Materials and methods

### 2.1 Model

**Fig. 1:**
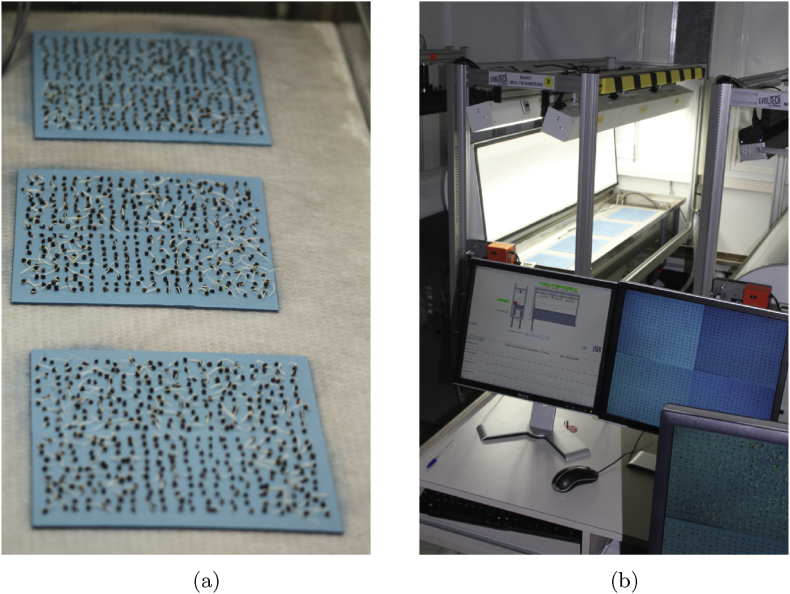
Germination rate monitoring system. (a) Seeds placed on a wet blotter. (b) Imaging system consisting of cameras and lights positioned above the seeds.

We examine data provided by the computer vision system shown in Fig. 1, where a camera captures top view images of seeds throughout the germination process. At the beginning of the experiment, approximately 100 dry seeds are placed on a moist blotter. As water gradually penetrates the seeds, the emergence of the seed radicle is detected using computer vision. The system then tracks and counts the number of germinated seeds. Currently, images are taken at fixed intervals of 2 ​h. Our goal is to optimize the sampling process in real-time via non-periodic sampling strategies.

In the current periodic sampling mode, germination rate measurement is performed every 2 ​h over the course of 168 ​h. The time vector, denoted $t={\left[t_{1},t_{2},\ldots ,t_{N}\right]}^{T}$, is defined as ***t*** ​= ​[0,2, *…*,168]^*T*^, where *N* ​= ​85 represents the total number of samples. The germination rate is denoted by ***y*** and modeled as

(1) y=g(t,θ)+η,

where ***η*** represents the measurement noise. We model this noise as a random vector following a multidimensional normal distribution of mean 0 and covariance **Σ**_*η*_, *i.e.* $\eta ∼N\left(0,\Sigma_{\eta }\right)$. Noise realizations are assumed to be independent. **Σ**_*η*_ is a matrix of size *N* with $\sigma_{\eta }^{2}$ on the diagonal and zero everywhere else. The growth model is chosen as

(2) $$g\left(t,\theta \right)=a\;e^{-b\;e^{-c\;t}},$$

which corresponds to the Gompertz model, well known in germination modeling, as highlighted by Tipton [9]. The model in Equation (2) depends on time *t* and the parameter vector ***θ*** ​= ​[*a*,*b*,*c*]^*T*^. Each of the three components (*a*, *b*, *c*) has biological interpretation: the parameter *a* corresponds to the final germination rate, *b* is related to the germination speed of the population and *c* determines the homogeneity of the population.

### 2.2 Adaptive sampling and Bayesian methods

Image capture and processing are energy-intensive activities. The lighting required for image capture can be intrusive for the germination process, which naturally occurs in obscurity within the soil [10]. Additionally, historical data must be kept for traceability, leading to significant storage requirements. To illustrate the scale of the problem addressed in this article, the variety testing office where the experiments were conducted has 10 vision systems similar to the one shown in Fig. 1. Each image is 10 Mo in size, with 4 cameras per system. Assuming continuous operation of all 10 vision systems, the total data output can reach up to 70 terabytes per year. To address this issue, we aim to reduce the number of measurements *N* by implementing an adaptive, non-periodic sampling method.

The basic idea is that the fixed-rate sampling approach often leads to redundant measurements, particularly when the germination process exhibits slow or predictable changes. To address this issue, we propose a method that estimates when to skip measurements. To achieve this, we propose to quantify and estimate, based on priors, the measurement uncertainty introduced by an additional data point before it is actually acquired. This estimation is updated every 2 ​h. If the measurement uncertainty exceeds a fixed threshold, a measurement is taken and saved; otherwise, it is skipped. The parameters of this method include the threshold for measurement uncertainty, *σ*_*T*_, the timeline ***t***, and its corresponding length *N*. The latter two parameters effectively define a minimum acquisition interval and a maximum experiment duration.

More mathematically, the adaptive sampling method iteratively constructs two vectors: the results of the adaptive sampling times, denoted $\tilde{t}$, and the corresponding saved measurements $\tilde{y}$. These vectors are defined as follows $\tilde{t}={\left[{\tilde{t}}_{1},{\tilde{t}}_{2},\ldots ,{\tilde{t}}_{N_{s}}\right]}^{T}$ and $\tilde{y}={\left[{\tilde{y}}_{1},{\tilde{y}}_{2},\ldots ,{\tilde{y}}_{N_{s}}\right]}^{T}$ where *N*_*s*_ corresponds to the sample size kept by our adaptive process. Some first measurements are needed to initiate the algorithm. The initial points (${\tilde{t}}_{1}=0h$, ${\tilde{t}}_{2}=8h$, and ${\tilde{t}}_{3}=16h$) were selected to ensure that the associated measure values (${\tilde{y}}_{1}=y_{1}$, ${\tilde{y}}_{2}=y_{5}$, ${\tilde{y}}_{3}=y_{9}$) were non zeros. The measurement uncertainty is denoted by $\sigma_{{\hat{y}}_{n}|\tilde{y}}$.

**Fig. 2:**
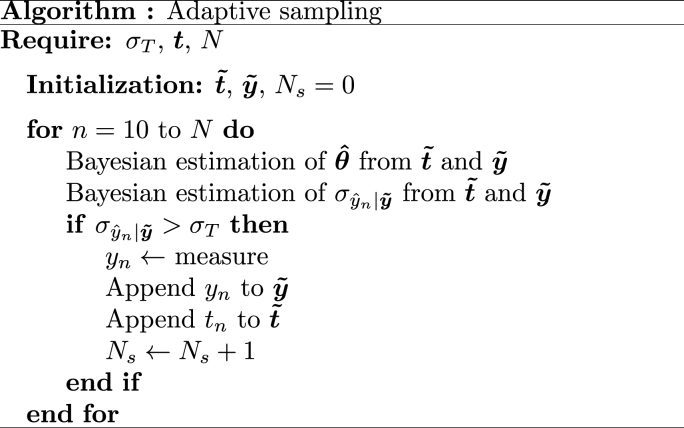
Adaptive sampling algorithm.

Bayesian analysis is chosen to take advantage of the germination model defined in Equation (2) and historical data. From a Bayesian perspective, this uncertainty corresponds to the predictive standard deviation of ${\hat{y}}_{n}|\tilde{y}$, which represents the predicted measure ${\hat{y}}_{n}$ at time *n* given the previous ones $\tilde{y}$ stored by the adaptive algorithm described in Fig. 2.

The computation of the measurement uncertainty $\sigma_{{\hat{y}}_{n}|\tilde{y}}$ is the critical step in the algorithm of Fig. 2. We propose Bayesian methods to estimate $\sigma_{{\hat{y}}_{n}|\tilde{y}}$, and update their values every 2 ​h, leveraging both prior knowledge and previous measurements. More, the Bayesian methods will also perform the estimation of the parameters $\Theta ={\left[\theta ,\sigma_{\eta }\right]}^{T}$ which are then used to estimate the germination rate $\hat{y}$ vector based on the function $g\left(t,\hat{\theta }\right)$, with the elements of $\hat{y}$ denoted as ${\left[{\hat{y}}_{1},{\hat{y}}_{2},\ldots ,{\hat{y}}_{N}\right]}^{T}$.

In this study, we tested five Bayesian methods to estimate the measurement uncertainty $\sigma_{{\hat{y}}_{n}|\tilde{y}}$: Importance sampling, MCMC, Gaussian process, extended Kalman filter and particle filter.

These five important techniques are used in statistical inference and machine learning. These techniques can be grouped into three categories: 1) Monte Carlo methods employed for approximating probability distributions, including MCMC, and for estimating expectations, such as IS. Indeed, the importance sampling is performed to estimate properties of a target distribution by sampling from a different distribution. The MCMC is a class of algorithms for sampling from probability distributions, particularly when those distributions are complex and difficult to sample from directly. It consists of constructing a Markov chain that converges to the target distribution. 2) Methods for non-parametric regression and classification as the Gaussian process. It provides a way to model functions directly, without assuming a specific parametric form. This makes it possible to evaluate the added value of explicit modeling by comparing model-based approaches to the non-parametric Gaussian process. 3) Methods used for sequential inference in dynamic systems include the extended Kalman filter and particle filters. Particle filters represent the probability distribution using a set of weighted particles and sequentially update the particle set as new data arrives. The extended Kalman filter performs by linearizing the non-linear dynamic system using the first-order Taylor series approximation and then applying the standard Kalman filter equations to the linearized system.

We describe how the uncertainty $\sigma_{{\hat{y}}_{n}|\tilde{y}}$ is calculated for each of the five Bayesian methods in the supplementary material. This allows readers interested in the phenotyping application to smoothly follow the results, while those interested in reproducing our method have access to all the necessary mathematical details.

### 2.3 Data and priors

For the experiments, we used the cumulative germination kinetics data from red clover accessions [11], which is available by following the link https://doi.org/10.57745/JECJUI. This diverse collection of red clover accessions is part of the Horizon EUCLEG project and is described in [12]. The experiments were conducted using the automated phenotyping platform PHENOTIC (SFR QUASAV, Angers) at 15 ​°C with a sampling time of 1 acquisition every 2 ​h. The detailed acquisition protocol is described in [3]. However, we emphasize that the growth model of Equation (2) is valid for any germination process. From the data set in [11], we selected three groups based on germination speed: fast, normal, and slow germination. This categorization is based on a standard seed certification metric, *t*_50_, which represents the time required to reach 50 ​% of germination time. It can be computed from the model parameters of Equation (2) as follows:

(3) $$t_{50}\left(\theta \right)=-\frac{1}{c}\ln\left(-\frac{1}{b}\ln\frac{50}{a}\right).$$

**Fig. 3:**
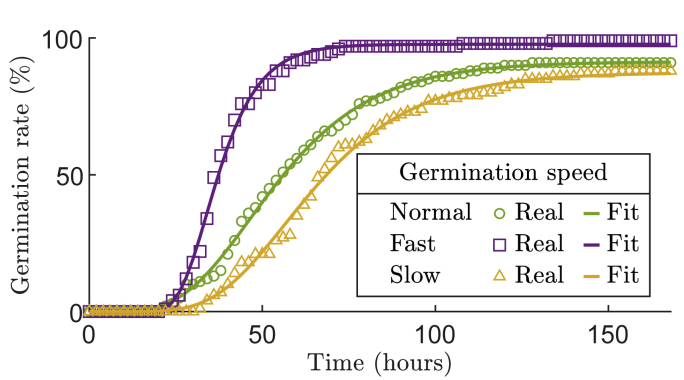
Real data example of the three sets fast, slow, and normal groups, along with their corresponding fits using Gompertz model.

Fast group is characterized by *t*_50_ values lower than first quartile, while the slow group has *t*_50_ values higher than the third quartile. The normal group has intermediate *t*_50_ values. An example of each germination speed is given in Fig. 3. Differences in germination speed can be due to genotypic variations, temperature differences or seed quality differences. In addition to real data, we generated simulated data to evaluate our adaptive sampling method. These simulated data are generated based on Equation (1), using parameter values of the Gompertz model ***θ*** and a centered Gaussian noise standard deviation *σ*_*η*_ estimated from the normal germination data.

The five Bayesian methods employed to estimate the measurement uncertainty $\sigma_{{\hat{y}}_{n}|\tilde{y}}$ of our adaptive sampling algorithm from Fig. 2 operates differently and are based on various priors. We provide the priors used by each method in Table 1 for ***θ***, the measurements *y* and the noise variance *σ*_*η*_. Priors are sorted in two types: initial value and distribution. All priors are based on the group of seed with normal speed. The train-test split among the normal data is random.

**Table 1:** Synthesis of prior knowledge by method.

Priors types		Methods	Priors
***θ***	**Initial value**	IS	–
		MCMC	Parameters average on normal training set
		GP	–
		EKF	Parameters average on normal training set
		SIR	–
	**Distribution**	IS	Parameters from normal training set
		MCMC	Uniform distribution (boundaries from normal training set)
		GP	–
		EKF	Gaussian distribution (parameters from normal training set)
		SIR	Uniform distribution (boundaries from normal training set)
*y*	**Distribution**	IS	–
		MCMC	–
		GP	Germination rates from normal training set
		EKF	–
		SIR	–
*σ*_*η*_	**Initial value**	IS	–
		MCMC	Estimation on normal training data
		GP	–
		EKF	–
		SIR	–
	**Distribution**	IS	Pre-determined parameters (average on normal training set)
		MCMC	Uniform distribution (boundaries from normal training set)
		GP	Pre-determined parameters (average on normal training set)
		EKF	Pre-determined parameters (average on normal training set)
		SIR	Uniform distribution (boundaries from normal training set)

### 2.4 Experiments and metrics

The five estimation methods presented in the previous section are compared in the context of adaptive sampling. The experiments were done using version 9.12.0.1884302 (R2022a) of the MATLAB software. The composition of the training and testing set is detailed in Table 2. Only normal germination data was used in the training set, *i.e.* as prior. This allows us to study the impact of deviation from this prior and reflects a real-world scenario in high-throughput phenotyping, where you cannot expect to have observed all possible configurations before automating measurements. This also corresponds to the timely issue linked with climate change, where the seed industry promotes seeds with fast germination at low temperature to enable earlier harvesting in the season, i.e. before chaotic extreme events (such as droughts and floods) which occur at the end of summer. The experiments were first performed on real and simulated data corresponding to normal germination speed to evaluate the performance in conditions corresponding to the priors used in training. We then investigated performance degradation when applying adaptive sampling methods to seeds with fast and slow germination speeds. For each experiment, 240 threshold values of *σ*_*T*_ between 0.1 and 12 were tested. This range is chosen to be large in order to cover compression rates from 0 to 1 over the training data.

**Table 2:** Training and testing sets compositions. Each set contains vectors of germination of length 85.

Speed	Use	Real data	Simulated data
Normal	Training	99	–
	Testing	30	20
Fast	Testing	30	–
Slow	Testing	30	–

Experiments were evaluated with metrics aligned to our goals, i.e. on-the-fly adaptive sampling leading to compression with minimal distortion. The compression ratio is defined as

(4) $$\text{Compression ​rate}=\frac{N_{s}}{N},$$

the length of the subsampled signal, *N*_*s*_, divided by the total length of the entire sample *N* which corresponds to the periodic sampling. If all samples are preserved, the signal is not compressed, and the compression ratio is equal to 1. Distortion measures the quality of the estimated germination rate $\hat{y}$ relative to the experimental full germination rate ***y***, as defined in Equation (1). As a distortion metric, we use the Mean Square Error (MSE) defined as follows

(5) $$\text{MSE}\left(\hat{y},y\right)=\frac{1}{N}\overset{N}{\underset{i=1}{\sum }}{\left({\hat{y}}_{i}-y_{i}\right)}^{2},$$

where here again we compare our reference, i.e. with zero *MSE* is the standard periodic sampling. The five estimation methods were also assessed for bias relative to the standard periodic sampling, with respect to global germination rate estimation through *t*_50_ and parameter estimation. We compared *t*_50_(***θ***) with its estimate $t_{50}\left(\hat{\theta }\right)$, as defined in Equation (3). The global bias metric is defined as $t_{50,\text{error}}=t_{50}\left(\theta \right)-t_{50}\left(\hat{\theta }\right)$. If the *t*_50_ error is positive, this means that the germination appears to be faster than it actually is. Conversely, if the *t*_50_ error is negative, it means that germination appears to be slower than it actually is. We compared the estimated germination rate model parameters [*a*, *b*, *c*], obtained using standard periodic sampling, with the estimates $\left[\hat{a},\;\hat{b},\;\hat{c}\right]$ obtained using adaptive sampling. The bias parameters are defined as follows: $\text{bias}\left(a\right)=a-\hat{a}$; $\text{bias}\left(b\right)=b-\hat{b}$; $\text{bias}\left(c\right)=c-\hat{c}$.

After experiments, the MSE, the *t*_50_ error, and the parameter bias are calculated for each instance in the test set. The reported metric value is the mean over all instances. The uncertainty *U* is computed under the assumption of a Gaussian distribution of the metric, with a 95 ​% confidence interval. The uncertainty *U* is defined as

(6) $$U=t_{95\%}\frac{\text{Std}}{\sqrt{N_{\text{ex}}}},$$

where *t*_95 ​%_ is the critical value from the Student's *t*-distribution for a 95 ​% confidence level, depending on the number of test instances, Std is the standard deviation of the corresponding metric, and *N*_ex_ is the number of instances in the test set.

## 3 Results

### 3.1 Distorsion compression trade-off

**Fig. 4:**
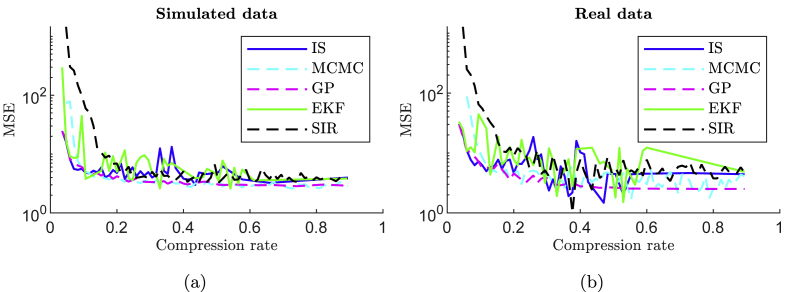
Estimation mean square error (MSE) of Equation (5) as a function of the compression ratio, obtained from simulated data (a) and real normal testing set (b) for the five tested Bayesian methods.

Fig. 4 shows the MSE as a function of compression to examine the compression-distortion trade-off. As expected, for both simulated and real data, the lower the compression ratio, the higher the distortion. The curves are L-shaped, and the optimal method is the one in which the corner of the L is closest to the graph origin. For both simulated and real data, IS, MCMC, and GP methods achieve similar lowest distortion and lowest compression. Regardless of the estimation method, we graphically determine that our adaptive sampling method is able to achieve a compression rate of 0.2, reducing the number of samples from 85 in the standard periodic sampling strategy to 17 with these best non-periodic sampling strategies.

### 3.2 Robustness and bias

We now assess robustness by analyzing the variability of the MSE and the estimation bias when adaptive sampling strategies are tested on different germination speeds. Table 3 presents the performance in terms of MSE at the compression rate of 0.2. For each test set, we applied 240 threshold values of *σ*_*T*_ and selected the instances that achieved a compression ratio of 0.2. Metrics are reported for each test set — Fast, Normal, Slow — to evaluate the impact of a shift in germination speed between prior knowledge (based on Normal germination speed) used in the Bayesian method and the test set.

**Table 3:** Distortion on real data set at a compression ratio of 0.2 (17 points).

	IS	MCMC	GP	EKF	SIR
	Log of mean square error				
**Normal**	1.62 ​± ​0.38	1.35 ​± ​0.17	1.53 ​± ​0.13	2.21 ​± ​0.68	1.96 ​± ​0.28
**Fast**	2.06 ​± ​1.30	1.34 ​± ​0.19	1.15 ​± ​0.14	2.20 ​± ​0.34	1.29 ​± ​0.48
**Slow**	4.20 ​± ​0.55	1.51 ​± ​0.20	1.84 ​± ​0.21	2.96 ​± ​1.16	3.15 ​± ​0.42

When tested on the normal data set, MCMC method achieves the lowest MSE among the five Bayesian estimation methods. When the Bayesian methods trained on the normal data set as a prior are tested on the fast data set, distortion increases only with the IS method. The GP method performs best on the fast data set, showing the lowest distortion, followed by the SIR and MCMC methods. When this experience is reproduced with the Slow data set as the test set, the MSE increases for all methods compared to testing on the normal data set. However, the MCMC method achieves the lowest MSE. Overall, at a compression ratio of 0.2, the MCMC method is the most robust to variations in germination speed, consistently delivering stable performance with low distortion.

Table 4 shows the global bias metric *t*_50,error_ and the parameters bias bias(*a*), bias(*b*) and bias(*c*). The global bias, measured as *t*_50,error_, shows the lowest results for MCMC across all conditions, highlighting its ability to withstand variation in germination speed. When it comes to estimating parameters, IS works best with normal data. The parameter bias increases quickly for both fast and slow data, showing that it depends a lot on the training data. GP is the most resistant to changes in speed in parameter estimation. This is true even though GP is not model-based, but rather data-centric.

**Table 4:** Bias analysis on real data set at a compression ratio of 0.2 (17 points).

	IS	MCMC	GP	EKF	SIR
	***t*_*50,error*_** (hours)				
**Normal**	0.49 ​± ​0.41	0.07 ​± ​0.18	−0.24 ​± ​0.12	0.21 ​± ​1.35	1.10 ​± ​0.30
**Fast**	−2.35 ​± ​2.67	0.13 ​± ​0.12	−0.12 ​± ​0.06	0.05 ​± ​0.48	0.25 ​± ​0.29
**Slow**	11.47 ​± ​4.26	0.36 ​± ​0.44	0.63 ​± ​0.34	−2.25 ​± ​3.44	4.38 ​± ​1.90

	**bias(*a*)**				
**Normal**	−0.11 ​± ​1.25	−0.81 ​± ​0.30	0.79 ​± ​0.52	−1.20 ​± ​1.68	−3.33 ​± ​0.90
**Fast**	0.25 ​± ​1.18	−1.15 ​± ​0.37	0.05 ​± ​0.29	0.62 ​± ​1.07	−1.01 ​± ​1.21
**Slow**	5.32 ​± ​4.10	−1.37 ​± ​0.56	−1.21 ​± ​1.25	−6.66 ​± ​11.83	−9.67 ​± ​2.68

	**bias(*b*)**				
**Normal**	0.41 ​± ​5.09	4.47 ​± ​1.95	−3.90 ​± ​2.82	−9.19 ​± ​15.22	11.23 ​± ​3.71
**Fast**	−7.07 ​± ​18.11	−5.69 ​± ​11.73	−2.47 ​± ​4.30	−35.44 ​± ​24.20	−3.08 ​± ​8.58
**Slow**	7.77 ​± ​2.76	3.94 ​± ​1.96	−0.45 ​± ​1.42	7.59 ​± ​12.22	23.78 ​± ​4.78

	**bias(*c*)**( ​× ​10^−2^)				
**Normal**	0.20 ​± ​0.25	0.34 ​± ​0.14	−0.24 ​± ​0.13	−0.27 ​± ​0.78	1.02 ​± ​0.24
**Fast**	−0.94 ​± ​1.66	0.21 ​± ​0.29	−0.06 ​± ​0.14	−1.60 ​± ​0.74	0.09 ​± ​0.47
**Slow**	1.28 ​± ​0.48	0.44 ​± ​0.14	0.06 ​± ​0.18	1.01 ​± ​1.82	2.37 ​± ​0.46

### 3.3 Computational cost

To complete the performance analysis of the tested adaptive sampling methods, we compare their computational cost. Table 5 presents the average computation time for each Bayesian estimation method in milliseconds.

**Table 5:** Average computation time per step by method in millisecond (ms). Computed on a *Dell Inc. Precision 7920 Tower* equipped with a processor *Intel(R) Xeon(R) Silver 4216 CPU @ 2.*10 ​GHz *2.*10 GHz *(2 processors)* over the normal training data.

Method	Average time per step (ms)
**IS**	2.30 ​± ​0.33
**MCMC**	2562.52 ​± ​442.61
**GP**	4.23 ​± ​4.53
**EKF**	0.01 ​± ​0.02
**SIR**	182.65 ​± ​19.63

The MCMC and SIR methods are the slowest, requiring 2.6 ​s and 0.2 ​s, respectively. Both methods rely on parameter sampling, which increases the computation time. The IS method, also based on parameter sampling, is faster taking only 2.3 ​ms. This difference comes from the number of parameter samples used: IS, MCMC, and SIR methods utilize 99, 10.000, and 2000 samples, respectively. The GP method requires 4.2 ​ms to estimate uncertainty. The EKF method is the fastest requiring just 0.02 ​ms per estimation. Although slow, the MCMC method, which provides the best results in terms of bias compression and distortion, remains compatible with on-the-fly computation since the biological speed of the germination processes remains much larger than the associated computation times.

## 4 Discussion

### 4.1 Monitoring

**Fig. 5:**
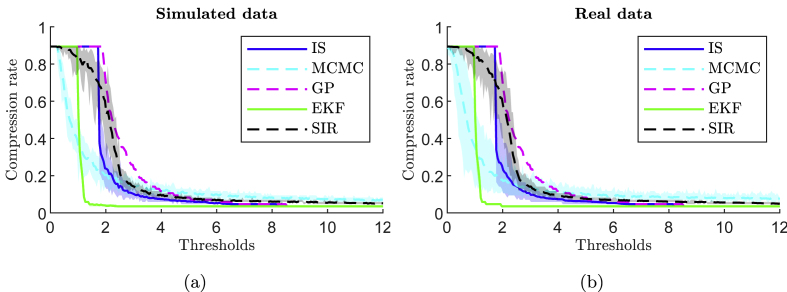
Average compression ratio as a function of threshold, obtained from 20 simulated data (a) and 30 real normal test data (b). The transparent colored band around the curve represents the range between the maximum and minimum values obtained over the iteration.

The threshold *σ*_*T*_ manages the trade-off between distortion and compression. According to the adaptivity of the method, the number of samples *N*_*s*_ and the MSE also depend on the previous measure. Fig. 5 shows the curves of the compression rate as a function of the threshold *σ*_*T*_ applied. The transparent colored band around the curve represents the range between the maximum and minimum values obtained during the iteration. As shown by the uncertainty, for SIR, MCMC and IS methods, a fixed threshold *σ*_*T*_ can lead to different compression rate i.e. different number of points *N*_*s*_. For GP and EKF methods, the number of points *N*_*s*_ can be perfectly controlled by the threshold *σ*_*T*_. However, Table 3 shows at fixed *N*_*s*_, uncertainty on MSE for GP and EKF. The distortion cannot be controlled perfectly. This issue needs to be addressed in future work to determine precisely if it is possible to constrain the MSE to a certain range.

### 4.2 Extension of the work

The proposed methodology offers an operational framework for efficient seed germination monitoring at low storage and computational costs. Further investigations could be conducted. While all the methodological implementation has been made for germination process, the methodological framework is, in principle, applicable for any temporal process. The Bayesian methods used for estimating the uncertainty $\sigma_{{\hat{y}}_{n}|\tilde{y}}$ designed for the nonlinear Gompertz model remain valid for other linear or nonlinear models of germination behavior. Two elements must be considered: 1) the choice of prior distributions for the model parameters and the range of variation for the hyperparameters of these distributions, and 2) the distribution of the observed data, i.e., the likelihood function. Consequently, the uniform prior distributions used in this study can be retained for other models, with an adjustment of the definition range of these distributions. It would be interesting to extend this approach to other phenotyping tasks such as sampling circadian cycles of leaves [13], seedling emergence [14] or the spread of pathogens. For circadian cycles specifically, the oscillations can fairly be assumed to be sinusoïdal so that they are based on 3 parameters (maximum amplitude, phase, average value). This would therefore not represent a significant change in terms of parameters to be learned by comparison with the parametric model used in this article. On the methodological side, other non-linear techniques, such as the Unscented Kalman Filter (UKF) [15], may improve performance for highly non-linear models. These directions could enhance the adaptability and robustness of the sampling method.

Another perspective to consider is the use of higher moments for adaptive sampling through the Gompertz germination model, rather than using the estimated mean and predictive standard deviation. Higher moments could be a fruitful area for future research.

## 5 Conclusion

In this work, we demonstrated the potential of online Bayesian adaptive sampling for monitoring germination rates. Various Bayesian methods were investigated to take benefit from the prior knowledge brought by previous measurements, historical data, and an expected non-linear model. These methods effectively reduced the frequency of measurements while maintaining accuracy, achieving a compression rate of 0.2 ​at reasonable computational cost.

This compression rate was achieved with the lowest distortion across IS, MCMC and GP methods. Regarding the distortion and estimation of the standard seed certification metric *t*_50_, MCMC proved to be the most robust method for adapting to variations in germination speed GP shows distortion close to that of MCMC. Over the different germination speeds, GP obtains the lowest parameter bias, ensuring reliable performance across different scenarios. Meanwhile, IS and GP offered substantially faster computational times.

## Author contributions

F. Mercier, N. Bouhlel, A. El Ghaziri, and D. Rousseau conceived and designed this work. J. Ly Vu, and J. Buitink produced the experimental data.F. Mercier, and N. Bouhlel implemented the sampling method. F. Mercier carried out the computations. F. Mercier, N. Bouhlel, A. El Ghaziri, and D. Rousseau conceived and interpreted the results. F. Mercier, N. Bouhlel, A. El Ghaziri, and D. Rousseau wrote and revised the manuscript. N. Bouhlel, A. El Ghaziri, and D. Rousseau supervised the work. All authors contributed equally to the writing of the manuscript.

## Funding

This research was funded by La Région des Pays de la Loire under the TANDEM program.

## Data availability

Data used in the article are table in.csv format containing raw germination along time available at the following repository https://doi.org/10.57745/JECJUI. [11].

## Declaration of competing interest

The authors declare that they have no known competing financial interests or personal relationships that could have appeared to influence the work reported in this paper.

## Figures and Tables

**Fig. 6:**
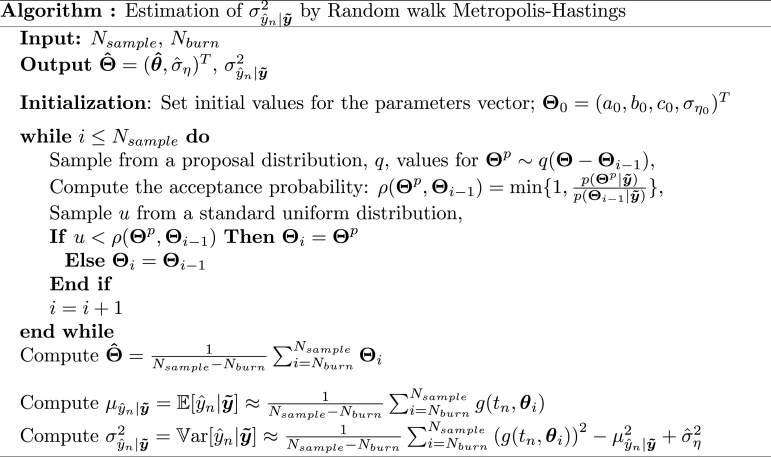
Random walk Metropolis-Hastings algorithm [18] for the estimation of $\sigma_{{\hat{y}}_{n}|\tilde{y}}^{2}$.

**Fig. 7:**
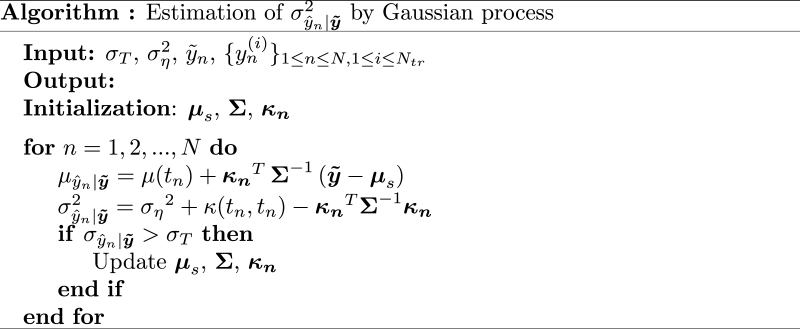
Gaussian process algorithm [16] for the estimation of $\sigma_{{\hat{y}}_{n}|\tilde{y}}^{2}$.

**Fig. 8:**
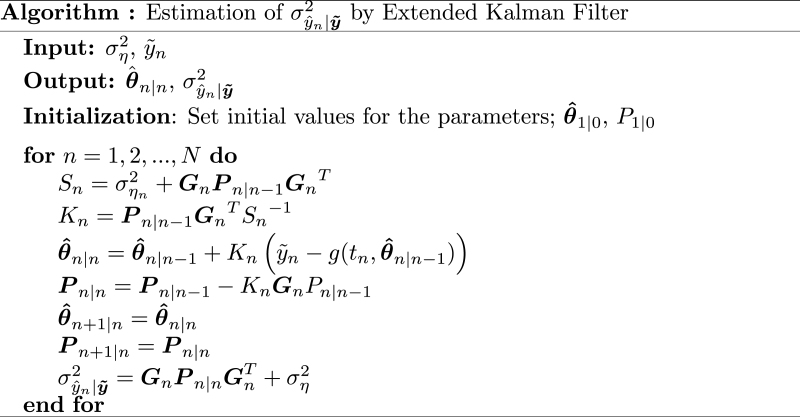
Extended Kalman filtering [21] for the estimation of $\sigma_{{\hat{y}}_{n}|\tilde{y}}^{2}$.

**Fig. 9:**
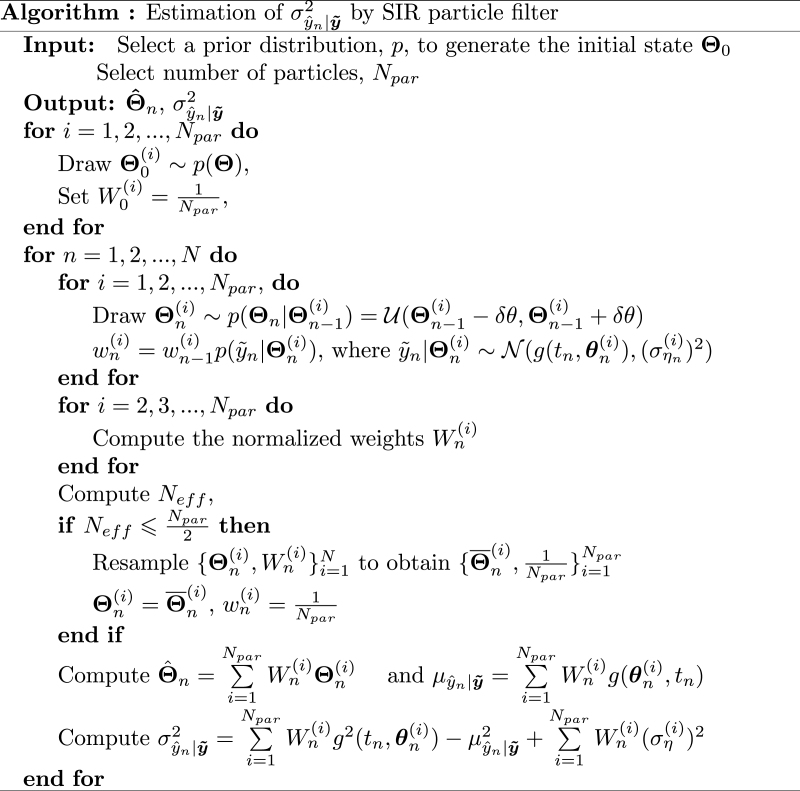
Sequential Important Resampling [16] for the estimation of $\sigma_{{\hat{y}}_{n}|\tilde{y}}^{2}$.

## Supplementary Materials

The supplementary material section includes the mathematical description for the five methods used to estimate the measurement uncertainty $\sigma_{{\hat{y}}_{n}|\tilde{y}}$ in the Algorithm of Fig. 2. The five methods are presented in the following order: Importance Sampling (IS), Monte Carlo Markov Chain (MCMC), Gaussian Process (GP), Extended Kalman Filter (EKF), Sequential Importance Resampling (SIR).

### Importance Sampling

Importance Sampling (IS) is a Monte Carlo method used to estimate properties of a distribution (like expectations or integrals) by drawing samples from a different proposal distribution [16,17]. We propose a variant of the IS method to assess uncertainty. The uncertainty $\sigma_{{\hat{y}}_{n}|\tilde{y}}$ is calculated using the following expectation-based formula:

(7) $$\sigma_{{\hat{y}}_{n}|\tilde{y}}^{2}=E\left[{\hat{y}}_{n}^{2}|\tilde{y}\right]-E{\left[{\hat{y}}_{n}|\tilde{y}\right]}^{2}.$$

The target distribution $p\left({\hat{y}}_{n}|\tilde{y}\right)$ is too complex for direct sampling. To address this challenge, IS introduces a simpler, more manageable proposal distribution that approximates the target distribution while being easier to sample from.

From the normal group, we build a training set. This set is denoted as ${\left\{\theta^{\left(i\right)}\right\}}_{1\leq i\leq N_{tr}}$, with *N*_*tr*_ = 99. These ***θ***^(*i*)^ serve as samples of the proposal distribution *p*(***θ***). The crucial step in IS is to reweight the samples drawn from the proposal distribution *p*(***θ***) to correct for the differences between the proposal and the target distributions. We use Bayes’ rules to incorporate the model function and the train set data into the weight. The target distribution can be expanded by marginalizing the joint distribution $p\left({\hat{y}}_{n},\theta \right)$ with respect to ***θ*** as

(8) $$p\left({\hat{y}}_{n}|\tilde{y}\right)=\int p\left({\hat{y}}_{n}|\theta \right)\;p\left(\theta |\tilde{y}\right)d\theta .$$

Computing this integral is very difficult, so it can be approximated by using samples from the posterior distribution $p\left(\theta |\tilde{y}\right)$.

Unfortunately, the latter is too complex to sample. By applying the IS principle, we make the prior distribution appear *p*(***θ***), which is easier to sample. Using Bayes’ rule, the posterior distribution can be expressed in terms of the likelihood $p\left(\tilde{y}|\theta \right)$ of the previous measurements and the prior distribution *p*(***θ***). The target distribution is then given by:

(9) $$p\left({\hat{y}}_{n}|\tilde{y}\right)=\int p\left({\hat{y}}_{n}|\theta \right)\;\frac{p\left(\tilde{y}|\theta \right)p\left(\theta \right)}{p\left(\tilde{y}\right)}d\theta .$$

Since the prior distribution *p*(***θ***) is under the integral, the Monte Carlo principle implies that the integral can be approximated by a sum over the ***θ***^(*i*)^ samples. The resulting expression is

(10) $$p\left({\hat{y}}_{n}|\tilde{y}\right)\approx \frac{1}{N_{tr}}\overset{N_{tr}}{\underset{i=1}{\sum }}p\left({\hat{y}}_{n}|\theta^{\left(i\right)}\right)\;\frac{p\left(\tilde{y}|\theta^{\left(i\right)}\right)}{p\left(\tilde{y}\right)}.$$

The probability density function (pdf) $p\left({\hat{y}}_{n}|\theta^{\left(i\right)}\right)$ is a normal distribution with a mean *g*(*t*_*n*_, ***θ***^(*i*)^) and a variance $\sigma_{\eta }^{2}$. The pdf $p\left(\tilde{y}|\theta^{\left(i\right)}\right)$ is a multivariate normal distribution with a mean of $g\left(\tilde{t},\theta^{\left(i\right)}\right)$ and a diagonal covariance matrix with diagonal element $\sigma_{\eta }^{2}$. It is worth mentioning that *σ*_*η*_ is unknown, and its value is estimated based on the training set. The marginal distribution $p\left(\tilde{y}\right)$ is computed using a similar approach as $p\left({\hat{y}}_{n}|\tilde{y}\right)$ by using ***θ***^(*i*)^ samples, and can be expressed as

(11) $$p\left(\tilde{y}\right)\approx \frac{1}{N_{tr}}\overset{N_{tr}}{\underset{i=1}{\sum }}p\left(\tilde{y}|\theta^{\left(i\right)}\right).$$

Once $p\left({\hat{y}}_{n}|\tilde{y}\right)$ is determined by Equation (10), it is then easy to compute the two expectations in Equation (7), namely, $E\left[{\hat{y}}_{n}^{2}|\tilde{y}\right]$ and $E\left[{\hat{y}}_{n}|\tilde{y}\right]$.

A by-product of IS sampling is the expected value of the measure defined as $\mu_{{\hat{y}}_{n}|\tilde{y}}=E\left[{\hat{y}}_{n}|\tilde{y}\right]$. In the final step of the adaptive sampling algorithm, the Levenberg–Marquardt algorithm (LMA) is used over the vector ${\left[\mu_{{\hat{y}}_{1}|\tilde{y}},\mu_{{\hat{y}}_{2}|\tilde{y}},\ldots ,\mu_{{\hat{y}}_{N}|\tilde{y}}\right]}^{T}$ to estimate $\hat{\theta }$. By introducing a simpler proposal distribution, *p*(***θ***), from which it is easier to sample, IS enables the efficient computation of the target distribution's expectations and statistical properties without directly sampling from it.

The convergence of importance sampling refers to how closely the estimator approaches the true value as the number of samples increases, and how efficiently it does so. In this study, we use a visual inspection of trace plots of the estimator as a diagnostic tool to determine the convergence and efficiency of an importance sampling estimator. This involves plotting the value of the importance sampling estimator as a function of the number of samples. If the estimator converges, the trace plot should stabilise around a particular value as the number of samples increases, becoming flatter. Conversely, a highly volatile trace that constantly drifts or shows large jumps indicates poor convergence or an unstable estimator.

### Markov Chain Monte Carlo

Markov Chain Monte Carlo (MCMC) is considered as a subclass of MC method. It is a powerful technique for dealing with probability distributions. It is primarily used for sampling from a target probability distribution. This is often the posterior distribution in Bayesian inference. Technically, MCMC constructs a Markov chain that, in the long run, converges to the target distribution. Common algorithms include Metropolis-Hastings and Gibbs sampling. With enough iterations, MCMC asymptotically converges to the true target distribution by providing samples from the latter.

The method assumes that the parameters *a*, *b*, *c*, and the noise standard deviation *σ*_*η*_ are independent random variables that follow prior distributions. The joint prior distribution of these parameters $\Theta ={\left[a,b,c,\sigma_{\eta }\right]}^{T}$ is given by *p*(**Θ**) = *p*(*a*)*p*(*b*)*p*(*c*)*p*(*σ*_*η*_). We choose simple prior distributions, where each parameter follows a uniform distribution within reasonable intervals: a∼U(0,100), b∼U(0,100), c∼U(0,10), and $\sigma_{\eta }∼U\left(0,10\right)$. In addition, the joint posterior distribution of the unknown parameters **Θ** is proportional to the product of the likelihood function (the probability density of the data) $p\left(\tilde{y}|\Theta \right)$ and the prior distribution, as follows

(12) $$p\left(\Theta |\tilde{y}\right)\propto p\left(\tilde{y}|\Theta \right)p\left(\Theta \right).$$

We apply the random walk Metropolis-Hastings algorithm [18] so that we can sample from the joint posterior distribution of the parameters **Θ**. The proposal distributions are chosen to be symmetric distributions, like a uniform distribution or a normal distribution. For the random walk Metropolis-Hastings algorithm, we use *p* index to refer to the proposal values of **Θ**^*p*^. We use the notation **Θ**_*i*_ to refer to a vector that will record the parameters considered in each step of the iteration. Proposal distributions for the parameters are: $a^{p}∼U\left(a_{i-1}-1,a_{i-1}+1\right)$, $b^{p}∼N\left(b_{i-1},1\right)$, $c^{p}∼+U\left(c_{i-1}-0.01,c_{i-1}+0.01\right)$ and $\sigma_{\eta }^{p}∼U\left(\sigma_{\eta_{i-1}}-0.1,\sigma_{\eta_{i-1}}+0.1\right)$ [8]. The Random walk Metropolis-Hastings algorithm is given in Fig. 6.

The number of sample used for Metropolis-Hasting sampler is denoted by *N*_*sample*_. It is important to notice that the acceptance probability depends on the ratio $r=\frac{p\left(\Theta^{p}|\tilde{y}\right)}{p\left(\Theta_{i-1}|\tilde{y}\right)}$. This ratio can be developed as follows

(13) $$r=\frac{p\left(\Theta^{p}|\tilde{y}\right)}{p\left(\Theta_{i-1}|\tilde{y}\right)}=\frac{p\left(\tilde{y}|\Theta^{p}\right)p\left(\Theta^{p}\right)}{p\left(\tilde{y}|\Theta_{i-1}\right)p\left(\Theta_{i-1}\right)}.$$

The new proposed value **Θ**^*p*^ is accepted with a probability *ρ*(**Θ**^*p*^, **Θ**_*i*−1_) = min{1, *r*}. If **Θ**^*p*^ is not accepted, the previous value **Θ**_*i*−1_ is kept. The generated samples are drawn from the posterior distribution $p\left(\Theta |\tilde{y}\right)$. We denote them by $\Theta_{\mathrm{sam}}=\left\{\Theta_{i},i=1,\ldots ,N_{\mathrm{sample}}\right\}={\left[\theta_{\mathrm{sam}},\sigma_{\eta_{\mathrm{sam}}}\right]}^{T}$ and we use them to compute uncertainty. So, to compute the predictive standard deviation $\sigma_{{\hat{y}}_{n}|\tilde{y}}$, the posterior predictive distribution, $p\left({\hat{y}}_{n}|\tilde{y}\right)$, is required. This distribution is expressed as a function of the posterior distribution of **Θ** given the stored measurement $\tilde{y}$ and the likelihood $p\left({\hat{y}}_{n}|\Theta \right)$ as follows

(14) $$p\left({\hat{y}}_{n}|\tilde{y}\right)=\int_{\Theta }p\left({\hat{y}}_{n}|\Theta \right)p\left(\Theta |\tilde{y}\right)d\Theta .$$

So, this integral is simply marginalizing out the parameters **Θ**. Unfortunately, it is often difficult to compute this integral, because the posterior density $p\left(\Theta |\tilde{y}\right)$ itself is intractable. For this reason, samples **Θ**_*sam*_ drawn from the posterior distribution of **Θ** given $\tilde{y}$ are used to approximate the posterior predictive distribution by using Monte Carlo draws, $p\left({\hat{y}}_{n}|\tilde{y}\right)=1/\left(N_{\mathrm{sample}}-N_{\mathrm{burn}}\right)\sum_{i=N_{\mathrm{burn}}}^{N_{\mathrm{sample}}}p\left({\hat{y}}_{n}|\Theta_{i}\right)$.

The burn-in period (*N*_*burn*_) is the initial part of the chain that is discarded prior to analysis. Specifically, given samples from the posterior distribution $\Theta_{i}∼p\left(\Theta |\tilde{y}\right)$, we can generate samples from the posterior predictive distribution ${\hat{y}}_{n_{i}}|\tilde{y}$ by randomly sampling from the distribution $p\left({\hat{y}}_{n}|\Theta_{i}\right)$ using the parameter draws. Since ${\hat{y}}_{n}|\Theta_{i}$ follows normal distribution $N\left(g\left(t_{n},\theta_{i}\right),\sigma_{\eta_{i}}^{2}\right)$, the expected value and the predictive standard deviation are given as follows

(15) $$\mu_{{\hat{y}}_{n}|\tilde{y}}=E\left[{\hat{y}}_{n}|\tilde{y}\right]\approx \frac{1}{N_{\mathrm{sample}}-N_{\mathrm{burn}}}\overset{N_{\mathrm{sample}}}{\underset{i=N_{\mathrm{burn}}}{\sum }}g\left(t_{n},\theta_{i}\right),$$

(16) $$\sigma_{{\hat{y}}_{n}|\tilde{y}}^{2}=V\text{ar}\left[{\hat{y}}_{n}|\tilde{y}\right]\approx \frac{1}{N_{\mathrm{sample}}-N_{\mathrm{burn}}}\overset{N_{\mathrm{sample}}}{\underset{i=N_{\mathrm{burn}}}{\sum }}{\left(g\left(t_{n},\theta_{i}\right)\right)}^{2}-\mu_{{\hat{y}}_{n}|\tilde{y}}^{2}+{\hat{\sigma }}_{\eta }^{2}.$$

where the set of estimated parameters $\hat{\Theta }$ is computed as follows

(17) $$\hat{\Theta }=\frac{1}{N_{\mathrm{sample}}-N_{\mathrm{burn}}}\overset{N_{\mathrm{sample}}}{\underset{i=N_{\mathrm{burn}}}{\sum }}\Theta_{i}.$$

There are several strategies that can be employed to control the convergence of the Markov chain constructed using the MCMC method. Various diagnostic measures can also be monitored to assist in this process. The most common approaches are:

• Increasing the number of iterations (chain length). Running the MCMC chain for longer periods gives more opportunity to explore the posterior distribution and converge to the stationary distribution. In this study, we choose to use *N*_*sample*_ = 10000.
• Choosing an adequate burn-in period. The initial samples of an MCMC chain are often influenced by the starting values and may not be representative of the target posterior distribution. Consequently, a longer burn-in period can help ensure that the subsequent samples are from the converged distribution. In this study, we choose *N*_*burn*_ = 7500.
• Visual inspection of trace plots. These plots show how each parameter's samples evolve over the iterations. A converged chain is characterised by a trace that forms a horizontal band without any obvious trends, drifts or periodic behaviour. We conclude that the MCMC chains have not converged when the trace shows trends, long flat regions, or high autocorrelation. Our study adopts this approach to controlling convergence.
• The Gelman–Rubin statistic (also known as the PSRF potential scale reduction factor) is a statistical test that quantifies the degree of convergence. This metric assesses the convergence of multiple MCMC chains that have been run with different starting points. It compares the variance between chains to the variance within chains for each parameter. Values close to 1 indicate that the chains have mixed well and are likely to have converged to the same target distribution. However, values significantly greater than 1 suggest that the chains are still exploring different parts of the parameter space and that longer runs are needed.

### Gaussian Process

Gaussian Process (GP) is a non-parametric approach used for regression and classification tasks. It provides a flexible framework for modeling distributions over functions, enabling uncertainty quantification in predictions [19]. The growth model, *g*(*t*_*n*_), is non-parametric. *g*(*t*_*n*_) replaces the Gompertz function in Equation (1). We assume *g*(*t*_*n*_) is a random variable and

(18) $$g\left(t_{n}\right)∼\text{GP}\left(\mu \left(t_{n}\right),\kappa \left(t_{n},t_{n^{\prime }}\right)\right).$$

A GP is defined by its mean $\mu \left(t\right)=E\left[g\left(t_{n}\right)\right]$ and its covariance function $\kappa \left(t_{n},t_{n^{\prime }}\right)=E\left[\left(g\left(t_{n}\right)-\mu \left(t_{n}\right)\right)\left(g\left(t_{n^{\prime }}\right)-\mu \left(t_{n^{\prime }}\right)\right)\right]$. The training set consists of *N*_*tr*_ signals, each containing *N* values corresponding to time points *t*_*n*_. We denote the training values as ${\left\{y_{n}^{\left(i\right)}\right\}}_{1\leq n\leq N,1\leq i\leq N_{tr}}$. The GP's mean and covariance are estimated over $\left\{y_{n}^{\left(i\right)}\right\}$ as follows:

(19) $$\mu \left(t_{n}\right)\approx \frac{1}{N_{tr}}\overset{N_{tr}}{\underset{i=1}{\sum }}y_{n}^{\left(i\right)},$$

(20) $$\kappa \left(t_{n},t_{n^{\prime }}\right)\approx \frac{1}{N_{tr}}\overset{N_{tr}}{\underset{i=1}{\sum }}\left(y_{n}^{\left(i\right)}-\mu \left(t_{n}\right)\right)\left(y_{n^{\prime }}^{\left(i\right)}-\mu \left(t_{n^{\prime }}\right)\right).$$

Theodoridis [16] details the regression method using GP modeling. He also derives the formula for estimating uncertainty:

(21) $$\sigma_{{\hat{y}}_{n}|\tilde{y}}^{2}={\sigma_{\eta }}^{2}+\kappa \left(t_{n},t_{n}\right)-{\kappa_{n}}^{T}\Sigma^{-1}\kappa_{n}.$$

Equation (21) presents the formula used to compute uncertainty. As with MC methods, *σ*_*η*_ must be known and is estimated from the training data. The kernel vector $\kappa_{n}={\left[\kappa \left(t_{n},t_{1}\right),\ldots ,\kappa \left(t_{n},t_{N_{s}}\right)\right]}^{T}$ contains the covariances between the prediction time point and the observed sample times. The matrix **Σ** represents the covariance matrix of the observations, with components defined as **Σ**_*j*,*k*_ = *κ*(*t*_*j*_, *t*_*k*_), where *j* and *k* range from 1 to *N*_*s*_.

The parameter vector $\hat{\theta }$ is estimated using the LMA over the vector of expected measurements, defined as $\left[\mu_{{\hat{y}}_{1}|\tilde{y}},\mu_{{\hat{y}}_{2}|\tilde{y}},\ldots ,\mu_{{\hat{y}}_{S}|\tilde{y}}\right]$, where *j* ranges from 1 to *N*_*s*_. The expected measurements are computed using the formula provided by Theodoridis:

(22) $$\mu_{{\hat{y}}_{n}|\tilde{y}}=\mu \left(t_{n}\right)+{\kappa_{n}}^{T}\;\Sigma^{-1}\;\left(\tilde{y}-\mu_{s}\right),$$

where $\mu_{s}={\left[\mu \left(t_{1}\right),\ldots ,\mu \left(t_{N_{s}}\right)\right]}^{T}$.

Standard metrics like MSE/RMSE are used to assess the uncertainty $\sigma_{{\hat{y}}_{n}|\tilde{y}}$ corresponding to the predictive standard deviation of ${\hat{y}}_{n}|\tilde{y}$. We can evaluate the MSE or RMSE of the GP's predictions on a test set. As the size of the training data increases, the error should decrease and converge to an irreducible noise level.

### Extended Kalman Filter

The Kalman filter and the Extended Kalman filter are both powerful tools for estimating the state of a system, but they differ significantly in their applicability and underlying assumptions. In one hand, Kalman filter is designed for linear systems with Gaussian noise. It uses a recursive algorithm to estimate the state of the system by combining predictions from a model with measurements [16]. On the other hand, extended Kalman filter (EKF) is designed for nonlinear systems. The principle of EKF is then to (1) linearize the nonlinear system around the current state estimate using a first-order Taylor series approximation, and (2) apply the Kalman filter equations to the linearized system [20,21]. It is worth mentioning that EKF is limited to Gaussian distributions, since it assumes Gaussian noise and approximates the posterior distribution as Gaussian.

To take advantage of the EKF method, we consider in our case that the parameters of the non-linear Gompertz model are unknown hidden state variables to be estimated. We assume that these are constant over time. The measurements/observations are represented by ${\tilde{y}}_{n}$. Technically, we perform a recursive inference for the parameters of the non-linear Gompertz model with the help of EKF from the set of all available measurements ${\tilde{y}}_{1:n}=\left\{{\tilde{y}}_{i},i=1,\ldots ,n\right\}$ up to time *n*. The state/observation relationship is defined by the following system

(23) $$\theta_{n}=\theta_{n-1},$$

(24) $${\tilde{y}}_{n}=g\left(t_{n},\theta_{n}\right)+\eta_{n},$$

where $\theta_{n}={\left[a_{n},b_{n},c_{n}\right]}^{T}$ and *η*_*n*_ is the noise realization as defined in Equation (1) at time *n*, and $\eta_{n}∼N\left(0,\sigma_{\eta }^{2}\right)$. In our context of adaptive sampling, ***θ*** is stationary, as formalized in Equation (23). Under this form, the method is also called recursive least square, as presented by Ifeachor [22].

Since the function *g*(*t*_*n*_, ***θ***_*n*_) is non-linear, then a local linearization of the equation is sufficient to describe the non-linearity. As detailed by Särkkä [23], this linearization around the current estimate is performed by Taylor series. The function *g*(*t*_*n*_, ***θ***_*n*_) is replaced by its Taylor series at the first order given as follows,

(25) $$G_{n}=\frac{dg\left(t_{n},\theta_{n}\right)}{d\theta_{n}}{|}_{\theta_{n}={\hat{\theta }}_{n|n-1}},$$

(26) $$=\left[1,\;-{\hat{a}}_{n|n-1}e^{-{\hat{c}}_{n|n-1}t_{n}},\;{\hat{a}}_{n|n-1}{\hat{b}}_{n|n-1}t_{n}e^{-{\hat{c}}_{n|n-1}t_{n}}\right]e^{-{\hat{b}}_{n|n-1}e^{-{\hat{c}}_{n|n-1}t_{n}}},$$

where ${\hat{\theta }}_{n|n-1}$ represents the estimate of ***θ*** at time *n* given observations up to time *n* − 1. Thus, we obtain a linear form of the system as follows

(27) $${\tilde{y}}_{n}=G_{n}\theta_{n}+\eta_{n}.$$

The EKF is based on this approximation, and $p\left(\theta_{n}|{\tilde{y}}_{1:n}\right)$ is approximated by a Gaussian distribution with mean ${\hat{\theta }}_{n|n}$ and covariance matrix *P*_*n*|*n*_ as follows $p\left(\theta_{n}|{\tilde{y}}_{1:n}\right)\approx N\left({\hat{\theta }}_{n|n},P_{n|n}\right)$. Through the recurrence principle, we can state also that $p\left(\theta_{n}|{\tilde{y}}_{1:n-1}\right)\approx N\left({\hat{\theta }}_{n|n-1},P_{n|n-1}\right)$, with the following relations representing the updated state estimate and updated covariance estimate,

(28) $${\hat{\theta }}_{n|n}={\hat{\theta }}_{n|n-1}+K_{n}\left({\tilde{y}}_{n}-g\left(t_{n},{\hat{\theta }}_{n|n-1}\right)\right),$$

(29) $$P_{n|n}=P_{n|n-1}-K_{n}G_{n}P_{n|n-1},$$

where *K*_*n*_ is the Kalman gain defined by

(30) $$K_{n}=P_{n|n-1}G_{n}^{T}S_{n}^{-1},$$

with

(31) $$S_{n}=\sigma_{\eta }^{2}+G_{n}P_{n|n-1}G_{n}^{T}.$$

The predicted state estimate and the predicted covariance estimate are given by ${\hat{\theta }}_{n+1|n}={\hat{\theta }}_{n|n}$ and ***P***_*n*+1|*n*_ = ***P***_*n*|*n*_. Fig. 8 provides a description of the EKF Algorithm. As mentioned in the algorithm for the initialization of the parameters, ***θ***_1|0_ = [88,25,0.06]^*T*^, *P*_1|0_ is a diagonal matrix with the values 1, 0.1 and 0.001 on the diagonal. For the standard deviation of the noise *σ*_*η*_, it is estimated based on the training data and the value is equal to 1.74.

The posterior predictive distribution of ${\hat{y}}_{n}$ given $\tilde{y}={\tilde{y}}_{1:n}$ is calculated by the following equation

(32) $$p\left({\hat{y}}_{n}|\tilde{y}\right)=\int_{\theta }p\left({\hat{y}}_{n}|\theta_{n}\right)p\left(\theta_{n}|\tilde{y}\right)d\theta_{n}.$$

Knowing that $p\left({\hat{y}}_{n}|\theta_{n}\right)=N\left(g\left(\theta_{n},t_{n}\right),\sigma_{\eta }^{2}\right)$ and $p\left(\theta_{n}|\tilde{y}\right)=N\left({\hat{\theta }}_{n|n},P_{n|n}\right)$, then the posterior predictive distribution follow normal distribution, $p\left({\hat{y}}_{n}|\tilde{y}\right)=N\left(\mu_{{\hat{y}}_{n}|\tilde{y}},\sigma_{{\hat{y}}_{n}|\tilde{y}}^{2}\right)$, where the parameters are given by

(33) $$\mu_{{\hat{y}}_{n}|\tilde{y}}=g\left(t_{n},{\hat{\theta }}_{n|n}\right),$$

(34) $$\sigma_{{\hat{y}}_{n}|\tilde{y}}^{2}=G_{n}P_{n|n}G_{n}^{T}+\sigma_{\eta }^{2}.$$

This follows from general properties of the Gaussian distribution [24].

It is worth noticing that accuracy degrades significantly when nonlinearities are severe or the system operates far from the linearization point. In these cases, the EKF can diverge and produce unreliable estimates if the linearization is poor. Recall that the EKF is limited to Gaussian distributions, since it approximates the posterior distribution as Gaussian. In order to assess the convergence of an EKF, it is necessary to evaluate the stability of the covariance matrix ***P***_*n*|*n*_ and the accuracy of the state estimates ${\hat{\theta }}_{n|n}$. A stable covariance matrix means that its elements should remain bounded over time. The state estimate is accurate when its bias is small.

### Sequential Importance Resampling Particle filters

Particle filters (PF), also known as sequential Monte Carlo (SMC) methods, are another powerful alternative techniques to EKF and popular approaches to deal with non-linear state-space models via probabilistic arguments [20]. PF are techniques of estimation based on simulation like MCMC. It is primarily used for sequential inference in dynamic systems, meaning that it naturally incorporates sequential information, which can be valuable for online parameter estimation or when data arrives sequentially [25]. Technically, the simulation-based method, PF, provides weighted samples called particles approximately distributed according to posterior distributions of the hidden states, given the values of the observation and sequentially updates the particle set as new data arrives [26].

The sequential importance sampling (SIS) algorithm forms the basis of most particle filters developed to date.

The various versions of particle filters proposed in the literature can be regarded as special cases of this general SIS algorithm. These special cases can be derived from the SIS algorithm by an appropriate choice of importance sampling density and/or modification of the resampling step [20].

Three particle filters are proposed in the literature and derived from the SIS algorithm: sampling importance resampling (SIR) filter [16], auxiliary sampling importance resampling (ASIR) filter [27,28] and regularized particle filter (RPF) [20]. In our study, we focused on the SIR filter. Specifically, SIR maintains a set of weighted samples that approximate the target distribution and sequentially improves the approximation by: (1) importance sampling, which involves drawing samples from a proposal distribution and weighting them according to their importance, (2) resampling by duplicating samples with high weights and discarding samples with low weights. The performance of SIR heavily relies on the choice of the proposal distribution and the resampling procedure, which reduce particle degeneracy problem (variance of the importance weights increases in time) [20,21,26]. In our context, we perform a sequential (recursive) Bayesian inference for the parameters of our non-linear model using SIR-PF. To do this, we treat the parameters of the model as the unknown hidden states. We assume that these are constant over time. The model has the following form,

(35) $$\Theta_{n}=\Theta_{n-1},$$

(36) $${\tilde{y}}_{n}=a_{n}e^{-b_{n}e^{-c_{n}t_{n}}}+\eta_{n},\;$$

where $\Theta_{n}={\left[a_{n},b_{n},c_{n},\sigma_{\eta_{n}}\right]}^{T}={\left[\theta_{n},\sigma_{\eta_{n}}\right]}^{T}$ and $\left\{\eta_{n},n\in N\right\}$ is an *i.i.d* measurement noise sequence that $\eta_{n}∼N\left(0,\sigma_{\eta }^{2}\right)$. The objective is to recursively estimate **Θ**_*n*_ at time *n* from measurements ${\tilde{y}}_{1:n}=\left\{{\tilde{y}}_{i},i=1,\ldots ,n\right\}$ up to time *n* and then estimate the predictive standard deviation $\sigma_{{\hat{y}}_{n}|\tilde{y}}^{2}$. To do this, the particle filter given by SIR algorithm generate a set of samples that approximate the posterior density $p\left(\Theta_{n}|{\tilde{y}}_{1:n}\right)$.

In order to develop the details of the algorithm, let $\left\{\Theta_{n}^{\left(i\right)},w_{n}^{\left(i\right)}\right\}$ denote a random measure that characterizes the posterior pdf $p\left(\Theta_{n}|{\tilde{y}}_{1:n}\right)$, where $\left\{\Theta_{n}^{\left(i\right)},i=0,\ldots ,N_{\mathrm{par}}\right\}$ is a set particle with associated weights $\left\{w_{n}^{\left(i\right)},i=1,\ldots ,N_{\mathrm{par}}\right\}$. The normalized weights corresponds to $W_{n}^{\left(i\right)}=\frac{w_{n}^{\left(i\right)}}{\sum_{i=1}^{N_{\mathrm{par}}}w_{n}^{\left(i\right)}}$. The posterior density at time *n* can be approximated as

(37) $$p\left(\Theta_{n}|{\tilde{y}}_{1:n}\right)=\overset{N_{\mathrm{par}}}{\underset{i=1}{\sum }}W_{n}^{\left(i\right)}\delta \left(\Theta_{n}-\Theta_{n}^{\left(i\right)}\right),$$

where *δ* stands for the Dirac measure. The weights are chosen by using the principle of importance sampling [25]. Thus, the weighted particles $\left\{\Theta_{n}^{\left(i\right)},i=0,\ldots ,N_{\mathrm{par}}\right\}$ are sampled easily from a proposal distribution, *q*(.), called the importance density [20], and consequently $\Theta_{n}^{\left(i\right)}∼q\left(\Theta_{n}|\Theta_{n-1}^{\left(i\right)},{\tilde{y}}_{n}\right)$. A popular choice for the proposal distribution is the prior

(38) $$q\left(\Theta_{n}|\Theta_{n-1}^{\left(i\right)},{\tilde{y}}_{n}\right)=p\left(\Theta_{n}|\Theta_{n-1}^{\left(i\right)}\right).$$

The prior density is chosen in our study as a uniform distribution, $p\left(\Theta_{n}|\Theta_{n-1}^{\left(i\right)}\right)=U\left(\Theta_{n-1}^{\left(i\right)}-\delta \theta ,\Theta_{n-1}^{\left(i\right)}+\delta \theta \right)$ where *δθ* is the bound of the particle update. That is $a_{n}=a_{n-1}^{\left(i\right)}+U\left(-5,5\right)$, $b_{n}=b_{n-1}^{\left(i\right)}+U\left(-1,1\right)$, $c_{n}=c_{n-1}^{\left(i\right)}+U\left(-0.01,0.01\right)$. For the initial prior, *p*(**Θ**_0_), a uniform distribution is also selected with limits given as follows: the minimum and the maximum values on the parameters of the training set: $a_{0}∼U\left(70,98\right)$, $b_{0}∼U\left(10,74\right)$, $c_{0}∼U\left(0.05,0.1\right)$. Consequently, the weighted particle update is given by the following

(39) $$w_{n}^{\left(i\right)}=w_{n-1}^{\left(i\right)}p\left({\tilde{y}}_{n}|\Theta_{n}^{\left(i\right)}\right).$$

The concept of resampling involves discarding particles with small weights while focusing on those with large weights. Among various resampling methods, the authors prefer systematic resampling [21] due to its simplicity of implementation. The effects of degeneracy can be reduced by resampling whenever a significant degeneracy is observed. In practice, a suitable measure of particle degeneracy is the effective number of samples *N*_*eff*_, also known as ESS, which is approximated by $N_{\mathrm{eff}}\approx 1/\sum_{i=1}^{N_{\mathrm{par}}}{\left(W_{n}^{\left(i\right)}\right)}^{2}$. Then, resampling is performed if *Neff* is below some threshold usually chosen as *N*_*par*_/2. The resampling step involves generating a new set ${\left\{{\bar{\Theta }}_{n}^{\left(i\right)}\right\}}_{i=1}^{N_{\mathrm{par}}}$ by resampling from $p\left(\Theta_{n}|{\tilde{y}}_{1:n}\right)$ and the weights are reset to 1/*N*_*par*_. Finally, the state variables (parameters) at each time instant are estimated as the weighted average of the generated particles, that is,

(40) $${\hat{\Theta }}_{n}=\overset{N_{\mathrm{par}}}{\underset{i=1}{\sum }}W_{n}^{\left(i\right)}\Theta_{n}^{\left(i\right)}.$$

Fig. 9 shows the description of the algorithm SIR-PF.

By proceeding the same way as the MCMC method, but replacing the samples drawn by MCMC by particles with their weights, we obtain the following results

(41) $$\mu_{{\hat{y}}_{n}|\tilde{y}}=\overset{N_{\mathrm{par}}}{\underset{i=1}{\sum }}W_{n}^{\left(i\right)}g\left(\theta_{n}^{\left(i\right)},t_{n}\right),$$

(42) $$\sigma_{{\hat{y}}_{n}|\tilde{y}}^{2}=\overset{N_{\mathrm{par}}}{\underset{i=1}{\sum }}W_{n}^{\left(i\right)}g^{2}\left(t_{n},\theta_{n}^{\left(i\right)}\right)-\mu_{{\hat{y}}_{n}|\tilde{y}}^{2}+\overset{N_{\mathrm{par}}}{\underset{i=1}{\sum }}W_{n}^{\left(i\right)}{\left(\sigma_{\eta }^{\left(i\right)}\right)}^{2}.$$

Controlling the convergence of a particle filter is crucial for ensuring its accuracy and reliability. To control and assess the performance of a particle filter, we can:

• Increase the number of particles (*N*_*par*_). As the number of independent samples (particles) increases, the empirical distribution formed by these particles will converge to the true underlying distribution. In this study, *N*_*par*_ = 2000.
• Monitor the ESS (*N*_*eff*_) over time. The ESS is a measure of the diversity of the particle set. A low ESS suggests that the filter may be suffering from particle degeneracy. We choose here the threshold for the ESS equal to *N*_*par*_/2.
• Use resampling techniques, triggered by the ESS, to eliminate low-weight particles and duplicate high-weight particles. The resampling technique corresponds to systematic resampling.
• Use metrics such as Mean Squared Error (MSE) and bias to check whether the estimates are converging towards the true values.
